# Supplementary material for: Effect of Precursors on the Electrochemical Properties of Mixed RuOx/MnOx Electrodes Prepared by Thermal Decomposition
Source: Materials (Basel). 2022 Oct 25;15(21):7489. doi: 10.3390/ma15217489 (PMC9655995; doi:10.3390/ma15217489)
Supplement: Supplementary file 1 [file materials-15-07489-s001.zip › materials-1937397-supplementary.pdf]

materials

Article

Effect of precursors on the electrochemical properties of mixed RuOx/MnOx electrodes prepared by thermal decomposition—Supplementary Materials

Elisabetta Petrucci[1,*], Francesco Porcelli[2], Monica Orsini[2], Serena De Santis[2] and Giovanni Sotgiu[2,*]

[1] Department of Chemical Engineering Materials Environment
[2] Department of Industrial, Electronic and Mechanical Engineering
* Correspondence: elisabetta.petrucci@uniroma1.it (E.P.); giovanni.sotgiu@uniroma3.it (G.S.)

Citation: Lastname, F.; Lastname, F.; Lastname, F. Title. *Materials* **2022**, *15*, x. https://doi.org/10.3390/xxxxx

Academic Editor: Firstname Lastname

Received: date
Accepted: date
Published: date

Publisher's Note: MDPI stays neutral with regard to jurisdictional claims in published maps and institutional affiliations.

Copyright: © 2022 by the authors. Submitted for possible open access publication under the terms and conditions of the Creative Commons Attribution (CC BY) license (https://creativecommons.org/licenses/by/4.0/).

1. SEM images at the lowest magnification available

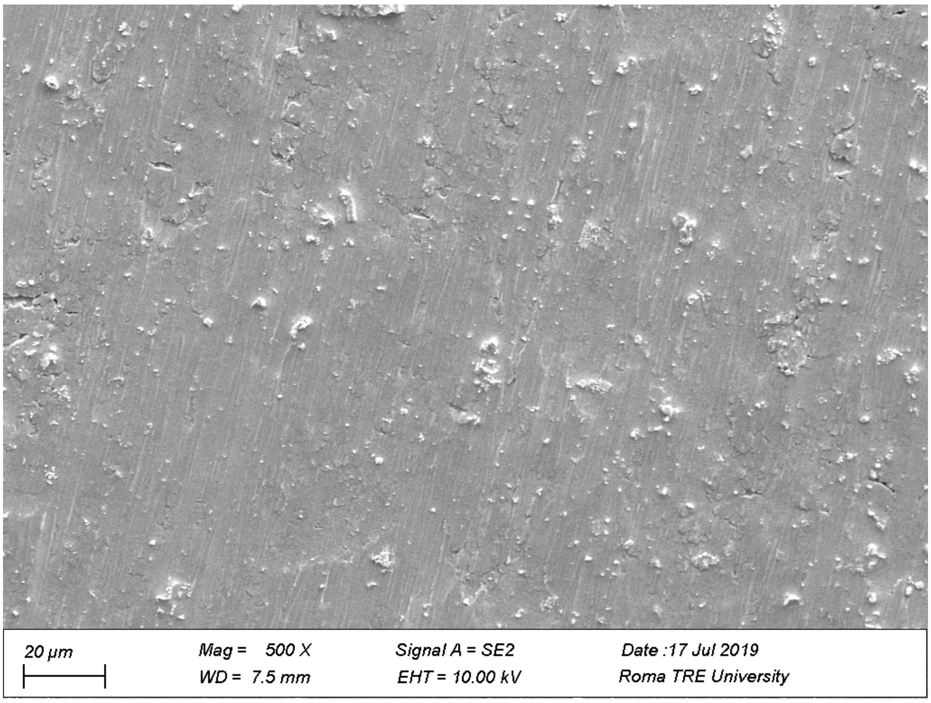

Figure S1. SEM image at larger scale for electrode E01.

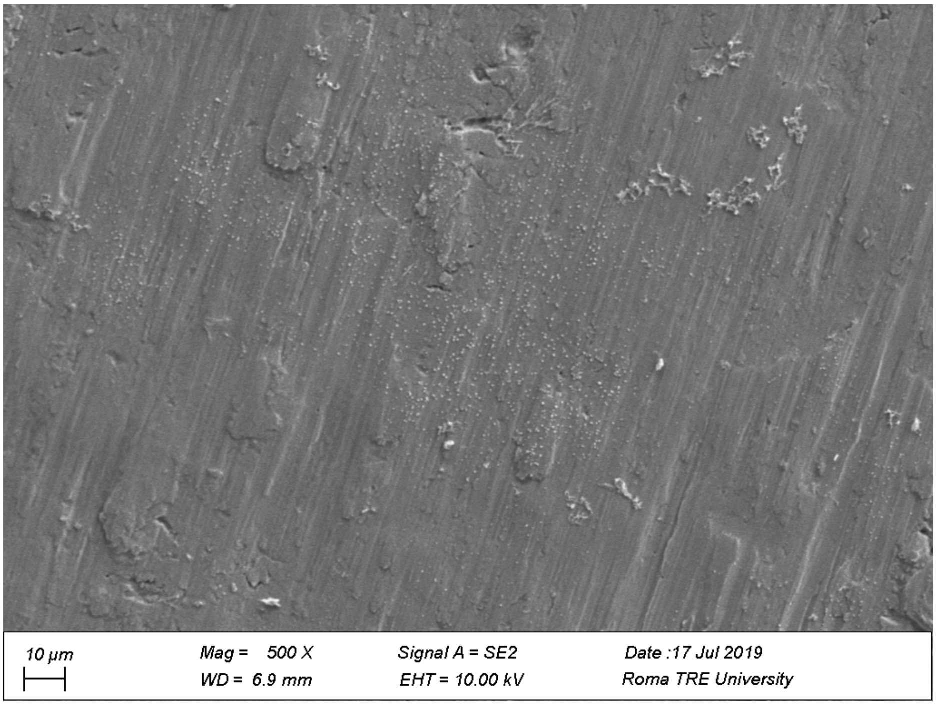

Figure S2. SEM image at larger scale for electrode E02.

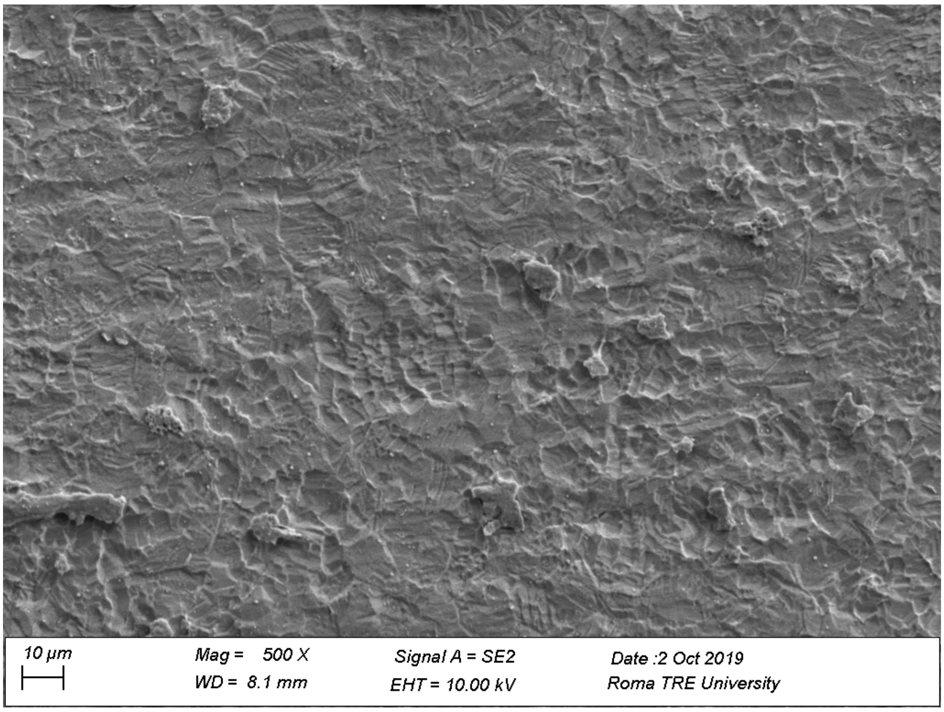

Figure S3. SEM image at larger scale for electrode E03.

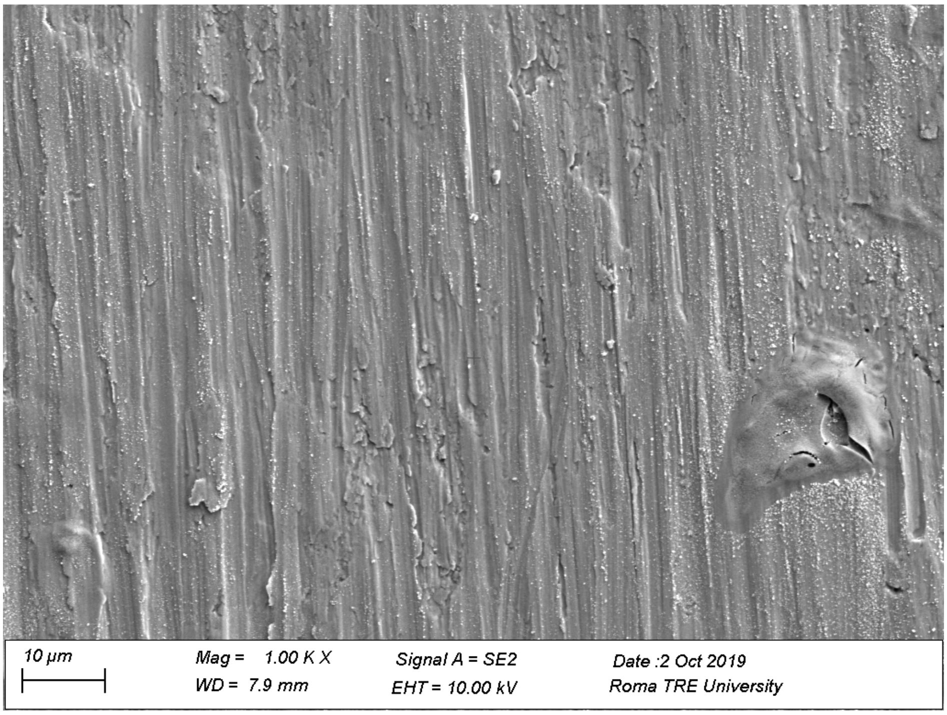

Figure S4. SEM image at larger scale for electrode E04.

2. EDS maps

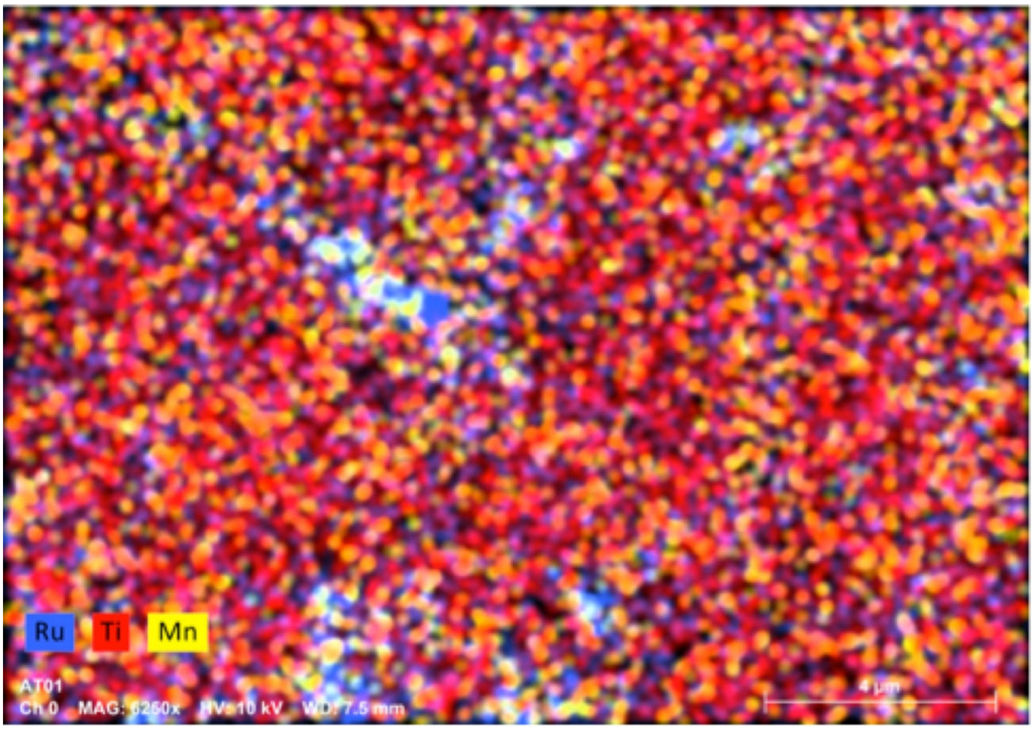

Figure S5. EDS map for electrode E01.

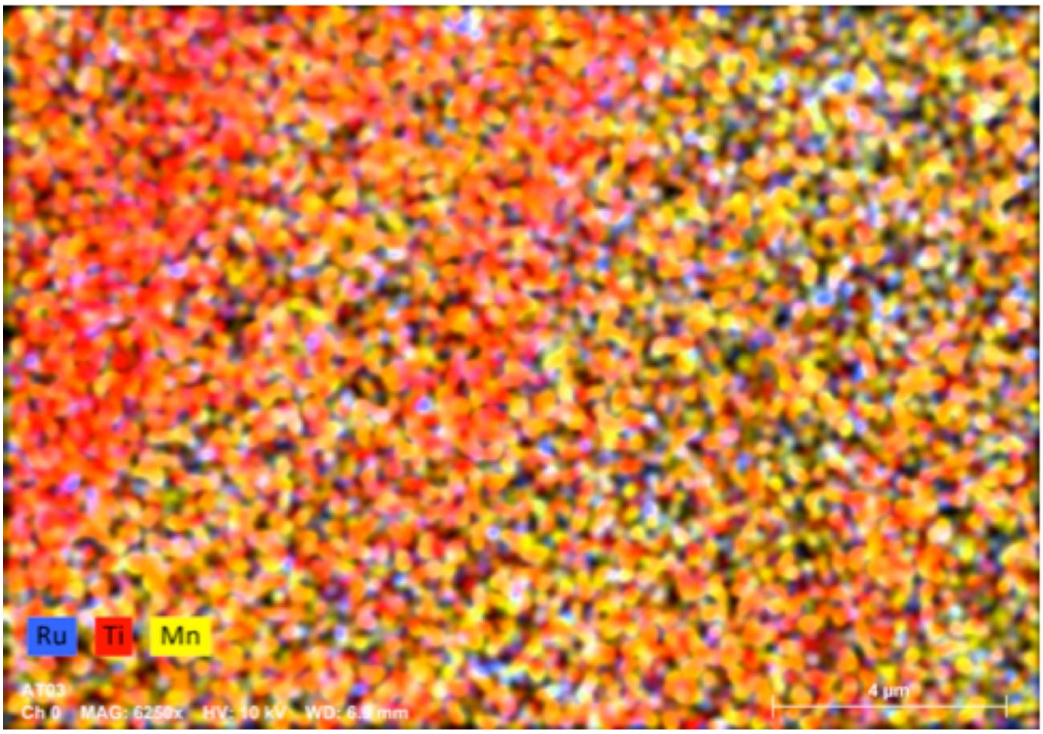

Figure S6. EDS map for electrode E02.

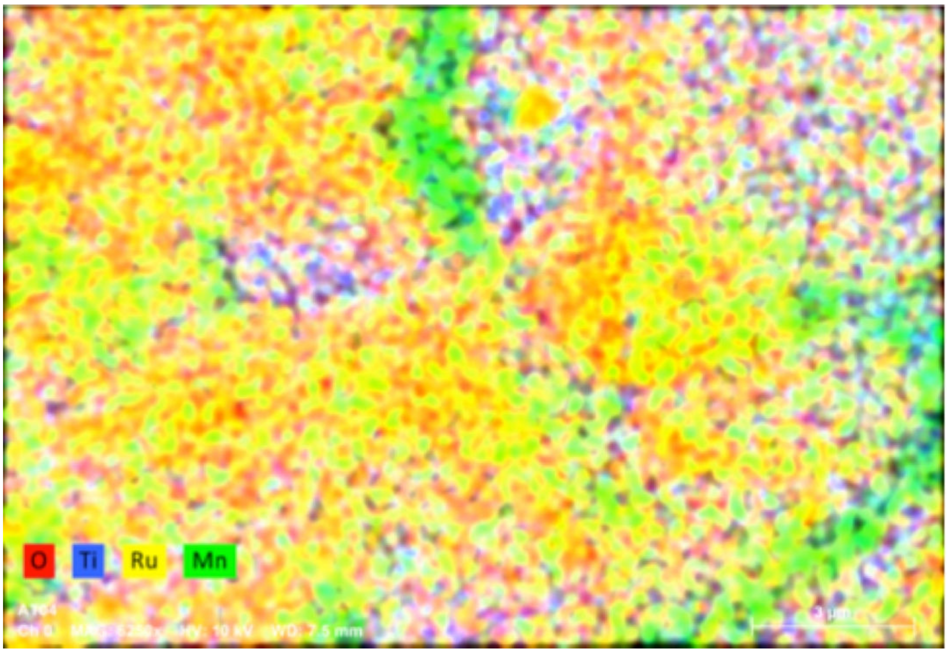

Figure S7. EDS map for electrode E03.

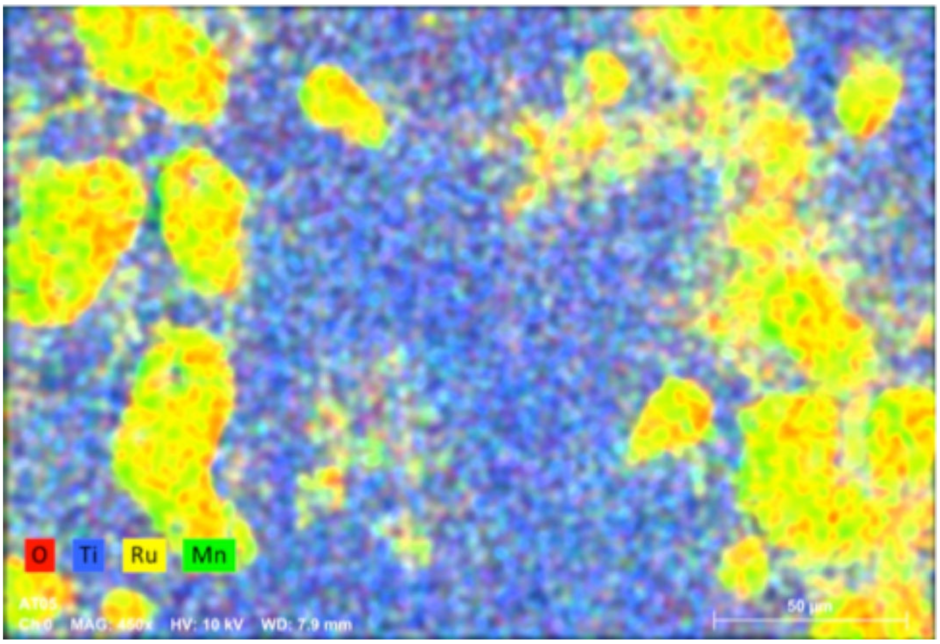

Figure S8. EDS map for electrode E04.

3. Electrochemical data - integration

An electrode prepared with the same procedure but starting from $RuCl_3$ and $Mn(NO_3)_2$ had been analyzed in a previous work [22]. Even if the solution in which it was analyzed is different from the current one, the extrapolated data are congruent and consistent with those presented in this publication.

Data extracted from these measurements are:

E_{corr} = 0.059 V

J0 = 3.69 µA cm^{-2}

q^+ (@ 50 mV s^{-1}) = 6.1 mC cm^{-2}
